# Patient safety culture and associated factors of regional public hospitals in Addis Ababa

**DOI:** 10.1186/s12913-024-11262-y

**Published:** 2024-07-12

**Authors:** Teshome Yayehrad, Yeneneh Getachew, Workineh Muluken

**Affiliations:** 1 Department of Public Health, MScHQ, Addis Ababa City Administration Health Bureau, P.O.Box 316, Addis Ababa, Ethiopia; 2 Institute for Healthcare Improvement, Addis Ababa, Ethiopia; 3 Department of Statistics, Wachemo University, P.O.Box 667, Hossana, Ethiopia

**Keywords:** Patient safety, Patient safety culture, Healthcare quality, Ethiopia

## Abstract

**Background:**

Patient safety culture is the result of individual and group values, attitudes, perceptions, competencies, and patterns of behavior that determine the commitment, style, and proficiency of health providers’ safety management. Globally, millions of adverse events occur annually, with a significant burden on low- and middle-income countries. The burden of injuries and other harm to patients from adverse events is likely one of the top 10 causes of death and disability worldwide. This study aimed to assess patient safety culture and its associated factors in regional public hospitals in Addis Ababa.

**Methods:**

An institution-based cross-sectional study was conducted among 494 healthcare professionals working at regional public hospitals in Addis Ababa. The data were collected using a pretested structured self-administered questionnaire from June 3 to July 30, 2023. The data were entered into Epi info version 7.2 and exported to SPSS version 26.0 for analysis. Binary logistic regression analysis was used to determine the associations between the patient safety culture (dependent variables) and socio-demographic factors, health care providers and system’s. Multicollinearity was checked using VIF, and the adequacy of the final model was assessed using the Hosmer and Lemeshow goodness-of-fit test.

**Result:**

Overall, 48.8% (95% CI: 44.3–53.1) of participants had a good patient safety culture, for a response rate of 93.3%. Factors significantly associated with patient safety culture, as identified through factor analysis, included having 6–10 years of experience (AOR = 1.81, 95% CI = 1.13–2.88), having more than 11 years of experience (AOR = 3.49, 95% CI = 1.27–9.56), reporting adverse events (AOR = 2.47, 95% CI = 1.37–4.45), participating in patient safety programs (AOR = 3.64, 95% CI = 1.91–6.92), and working in obstetrics and pediatric wards (AOR = 0.47, 95% CI = 0.23–0.94) and (AOR = 0.21, 95% CI = 0.097–0.44), respectively.

**Conclusion:**

The overall level of patient safety culture in regional public hospitals was low (< 75%). Factors such as having 6 or more years of experience, reporting adverse events, participating in patient safety programs, and working in obstetrics and pediatric wards were significantly associated with patient safety culture.

**Supplementary Information:**

The online version contains supplementary material available at 10.1186/s12913-024-11262-y.

## Introduction

Patient safety refers to the prevention of harm to patients during healthcare, reducing the risk of unnecessary harm to an acceptable minimum [1]. An adverse event is an incident that causes preventable harm to a patient, while an error is a failure to carry out a planned action correctly or the application of an incorrect plan [2].

Patient safety culture is the result of individual and group values, attitudes, perceptions, competencies, and patterns of behavior that determine the commitment, style, and proficiency of health providers’ safety management [3].

To improve patient safety, understanding of patient safety culture is critical for improving undesirable workforce attitudes, values and behaviors such as miscommunication on adverse events and maintaining the quality of care [4]. Conducting a safety culture assessment allows organizations to grasp healthcare professionals’ perceptions and attitudes related to safety, enabling the identification of issues that may lead to errors [5].

Despite global efforts to reduce patient harm, it remains a significant public health challenge, ranking among the leading causes of death and disability worldwide [6]. Every year, approximately 421 million people are hospitalized worldwide, 42.7 million of whom experiencing adverse events during their stay. It is estimated that 1 in 10 hospitalized patients suffer harm, with at least 50% of these cases being preventable [1]. Adverse events and resulting harm to patients are among the top 10 causes of death and disability globally, and are comparable to tuberculosis and malaria. Low- and middle-income countries bear the majority of this burden, with 134 million healthcare-associated adverse events occurring annually in hospitals, contributing to 2.6 million deaths [7]. Adverse events resulting from a deficient patient safety culture impose a significant burden on patients and the healthcare system, necessitating substantial financial resources for compensating for preventable medical errors [6].

The World Health Organization highlights the substantial impact of unsafe clinical care on patient mortality [8]. Unsafe medical practices pose significant threats to patient safety. The leading causes of patient harm and mortality include medication errors, healthcare-associated infections, unsafe surgical procedures, hazardous injection practices, improper transfusions, radiation errors, sepsis, and venous thromboembolism (blood clots) [1]. A study carried out in Ethiopia on the incidence and root causes of medication errors by anesthetists showed that more than half (64.4%) of participants experienced at least one drug administration error [9]. Adverse events caused by not only unsafe medical practice but also systemic failures including medical equipment deficiency, inadequate staffing and communication errors [10, 11].

A study carried out in Brazil’s three maternity hospitals’ gynecology and obstetrics departments and primary healthcare units revealed that the general scores of safety culture were 40.7% and 49.9%, respectively [12, 13]. Similarly, a study conducted in China among ECMO teams in the emergency department showed that the overall level of patient safety culture was 47.6% [14].

A study in Ethiopia carried out in Dessie, Jimma zone, Addis Ababa, and Bale zone hospitals showed that the overall level of patient safety culture was 44.8%, 46.7%, 44% and 44% respectively [15–18].

Many factors can be associated with patient safety culture. One study showed that patient safety culture is influenced by teamwork across hospital units, feedback and communication about errors, underreporting of adverse events, working units, hospital type, management support, and the number of years worked in the current hospital [12, 15, 18, 19].

A poor patient safety culture in healthcare has led to a rise in adverse events and has a detrimental effect on patient safety and healthcare quality, but it also diminishes patients’ quality of life, increases readmission rates, and negatively affects staff morale and competence [5]. However, there is still a gap in patient safety culture in the country and study area. In Ethiopia, the National Health Care Quality and Safety Strategy (NQSS) (2021–2025) are best suited for improving patient safety culture. The current state of patient safety culture has not yet been measured in this study area-a gap that needs to be addressed through the National Health Care Quality and Safety Strategy.

Therefore, this study aimed to assess patient safety culture and identify factors associated with regional public hospitals in Addis Ababa, Ethiopia.

## Methods

### Study design, setting and period

An institutional-based cross-sectional study was conducted from June 3, 2023, to July 30, 2023, in Addis Ababa regional hospitals. Addis Ababa is the capital city of Ethiopia, and has an area of 530 km2. It was estimated that 6.6 million people resided in the city in 2017, which was calculated based on the 2007 population enumeration with a 3.8% yearly growth rate. Addis Ababa has an area of 527 km² with an altitude of 2,355 m.a.s.l. It has 11 sub cities and different government health facilities, including 15 public hospitals in Addis Ababa, and six hospitals under the administration of the Addis Ababa Regional Health Bureau (AARHB), nine of which are under the federal ministry of health.

Two public hospitals under the Addis Ababa Regional Health Bureau, Zewuditu Memorial Hospital and Ras Desta Memorial Hospital, were included in the study area. Zewuditu Memorial Hospital is a general hospital located in Kirkos Sub city, around Filweha Spa. It has a total of 1,137 staff members, with 317 supportive staff members and 818 healthcare professionals, 415 of whom are nurses. Ras Desta Memorial Hospital was initially established by Italian Catholic missionaries on August 13, 1932 (1924 E.C). Currently, the hospital has a medical facility that provides healthcare services to both inpatients and outpatients. It has total of approximately 918 employees, 569 of whom are health professionals, and 229 of whom are nurses.

### Source population

All healthcare professionals who worked in regional public hospitals in Addis Ababa were the source population.

### Study population

All selected healthcare professionals who worked in selected regional public hospitals in Addis Ababa during the time of data collection.

### Sample size

The sample size was determined using OpenEpi version 2.3 for a single population proportion. The following assumptions were considered: an estimated proportion (p) of 44.8% from a similar study conducted at public hospitals in Dessie town, Northeast Ethiopia in 2021 [18], a margin of error of 5%, and a confidence level of 95%. Based on these findings, the sample size was determined to be 299 (*n* = 299). Considering the design effect of 1.5 due to the two-stage design, the sample size was calculated as 299*1.5 = 449. Additionally, a nonresponse rate of 10% was taken into account, resulting in a final sample size of 449 + 45 = 494.

The sample size calculated for the second objective was smaller than the sample size calculated for the first objective i.e. 494. Therefore, the final sample size for this study was determined to be 494 (*n* = 494).

### Sampling technique and procedure

**Fig. 1:**
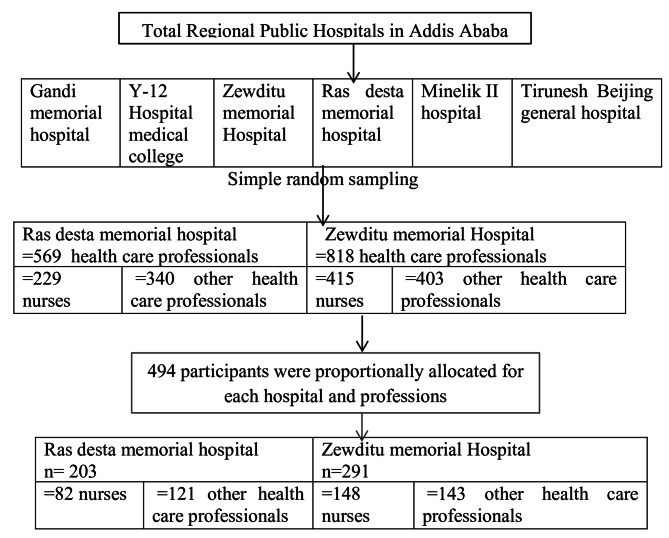
Flow chart of participant enrolment for the study

Simple random sampling with the lottery method was used to select two regional public hospitals in Addis Ababa from a total of six regional hospitals. The two selected hospitals were the Ras Desta and Zewuditu Memorial hospitals. The sample size was allocated proportionally to each selected hospital and was also proportionally allocated between nurses and other healthcare professionals, i.e. Midwife, Physician, Pharmacist, Laboratory, Anesthesiology, Radiology, Psychiatry, and Administrative at each hospital. Finally, simple random sampling was used to select healthcare providers from among both nurses and other healthcare professionals in the selected hospitals. The list of study participants was obtained from the human resource office of each hospital. (see Fig. 1)

### Data collection tool and procedure

The Hospital Survey on Patient Safety Culture (HSOPSC) is a standardized, structured, self-administered questionnaire used to collect quantitative data on patient safety culture. The questionnaire consists of 42 items grouped into 12 dimensions or composites: ‘Teamwork across units’ (4 items), ‘Teamwork within units’ (4 items), ‘Communication openness’ (3 items), ‘Feedback and communication about errors’ (3 items), “Handoffs and transitions” (4 items), “Frequency of events reported” (3 items), “Overall perceptions of patient safety” (4 items), “Organizational learning-continuous improvement” (3 items), “Management support for patient safety” (3 items), “Nonpunitive response to error” (3 items), “Staffing” (4 items), and “Supervisor/manager expectations and actions promoting safety” (4 items). Each item in the questionnaire is assessed using a 5-point Likert scale ranging from 1 (“Strongly disagree”) to 5 (“Strongly agree”) or from 1 (“Never”) to 5 (“Always”) for frequency. The questionnaire includes both positively and negatively worded items for agreement and frequency, as well as demographic characteristics of the professionals and other factors [20]. The questionnaire was originally in English and was then translated to Amharic and returned to English by another translator to ensure message consistency. The translation was reviewed by professional experts. This paper-based questionnaire, together with the participant information statement, was distributed to the selected participants by a person recruited for this purpose. The questionnaire required approximately 10–15 minutes to complete. Before beginning the actual data collection, a pretest was conducted with 5% of the sample at Yekatit 12 Hospital Medical College to check the validity and consistency of the Amharic translated version of the questionnaire.

### Operational definitions

#### A good patient safety culture

is indicated by a mean score > = 75% of the Hospital Survey on Patient Safety Culture (HSOPSC) questions [18].

#### Poor patient safety culture

mean score *<* 75% of Hospital Survey on Patient Safety Culture (HSOPSC) questions [18].

### Study variables

The dependent variable was patient safety culture and the independent variables were sociodemographic factors, health care provider characteristics and health care system-related factors.

### Data quality control

The data collection tool used in this study has been widely used around the world, including by researchers in Ethiopia [16–18]. The instrument was pretested to ensure the content and clarity of the questionnaires. A one-day training session was conducted by the principal investigator for five BSC nurses and two supervisors, covering the basics of the questionnaire and how to use it correctly. The data collection procedure was supervised by a supervisor, who checked for completeness and logical consistency at the end of each day and provided immediate feedback during the data collection process.

### Data analysis and management

The data were cleaned, stored, and exported to SPSS version 26.0 for analysis after being coded and entered into Epi Info version 7.2. For negatively worded items of the HSOPSC, reverse coding was performed so that a higher score would indicate a more positive response. The data are summarized using frequency and percentage for categorical covariates, and the numerical variables are summarized as medians and interquartile ranges (IQR).

Binary logistic regression analyses were used to determine the associations between the dependent variables and the independent variables. Bivariate analysis was performed to calculate unadjusted odds ratios (ORs) and to screen for potentially significant independent variables. Variables with a *p*-value ≤ 0.25 were included in the multivariable binary logistic regression model. The odds ratio (OR), *p*-value, and 95% Confidence interval (CI) for odds ratio (OR) were used to test the significance and interpret the results. Variables with a *p*-value ≤ 0.05 were considered statistically significant.

The basic assumptions of the binary logistic regression model, namely multicollinearity and outliers, were checked using VIF and box plots, respectively. The adequacy of the final model was assessed using the Hosmer and Lemeshow goodness-of-fit test, where a *p*-value > 0.05 indicated that the final model fit the data well.

## Results

### Sociodemographic variables

From a total of 494 samples, information was collected from 461 healthcare professionals, resulting in a response rate of 93.3%. More than half of the participants were female (238, 51.8%) and 252 (54.7%) of the participants held a bachelor’s degree. The median age was 29 year interquartile range (IQR) [28–32]: and 238 (51.6%) of the participants were under the age of 29 years (see Table 1).

**Table 1:** Socio-demographic characteristics among health care professionals (*N* = 461)

Variables	Category	Frequency	Percent (%)
age	<=29	238	51.6
	30–34	166	36.0
	>=35	57	12.4
sex	Male	222	48.2
	female	239	51.8
Educational status	Bachler’s degree	252	54.7
	Medical Doctors	62	13.4
	Master’s degree	125	27.1
	others	22	4.8

### Facility and work related characteristics

Nearly half of the participants, 47.7% (220), were nurses. More than half, 57.5% (266), had less than five years of work experience, and only 19.7% (91) reported events. Over 90% of the participants had direct contact with patients. More than half, 57.5% (265), were from Zewditu Hospital. Approximately 28.9% (three out of ten) had received patient safety training, and 16.1% (74) were participating in a patient safety program (as shown in Table 2).

**Table 2:** Facility and wok related characteristics among health care professionals (*N* = 461)

Variables	Category	Frequency	Percent (%)
Work place	Zewditu	265	57.5
	Ras Desta	196	42.5
Patient safety training	no	328	71.1
	yes	133	28.9
Participate in patient safety training program	no	387	83.9
	yes	74	16.1
Adverse event reports	no	370	80.3
	yes	91	19.7
Work experience	<=5	266	57.7
	6–10	166	36.0
	>=11	29	6.3
Total hours work per week	20–39	53	11.5
	40–59	170	36.9
	60–79	95	20.6
	>=80	143	31.0
Position	nurse	220	47.7
	midwife	46	10.0
	physician	67	14.5
	pharmacist	35	7.6
	laboratory	28	6.1
	administrative	25	5.4
	other	40	8.7
Working area/unit	Medical	89	19.3
	Surgery	64	13.9
	Obstetrics	69	15.0
	Paediatrics	76	16.5
	Emergency department	42	9.1
	ICU	31	6.7
	Pharmacy	36	7.8
	Laboratory	28	6.1
	others	26	5.6
Direct contact to patients	no	25	5.4
	yes	436	94.6

### Level of patient safety culture

Almost half, 228 (48.8%) with a 95% CI of 44.3–53.1, of the professionals reported/perceived that a good patient safety culture existed in their institution.

Regarding the average percentage of positive patient safety culture scores on dimensions, teamwork within units scored 77.7%; other dimensions’ scores ranged from 44.7 to 55.3% (see Table 3).

**Table 3:** Patient safety culture dimensions of regional public hospitals in addis ababa, Ethiopia (*N* = 461)

S.no	Patient safety culture dimensions	Numbers of items	Positive patient safety score %
1	Teamwork Within Units	4	77.7
2	Teamwork Across Units	4	54.4
3	Supervisor/Manager Expectations and Actions Promoting Patient Safety	4	52.5
4	Communication Openness	3	54.7
5	Feedback and Communication About Error	3	53.4
6	Frequency of Events Reported	3	44.7
7	Handoffs and Transitions	4	55.3
8	Management Support for Patient Safety	3	54.9
9	Staffing	4	46.0
10	Organizational Learning—Continuous Improvement	3	50.1
11	Overall Perceptions of Patient Safety	4	52.3
12	Non-punitive Response to Error	3	48.6

### Factors associated with patient safety culture

From the bivariate analysis, it was found that work experience, adverse event reports, work area/unit, educational status, patient safety training, and participation in patient safety programs were significantly associated with the level of patient safety culture in regional public hospitals at a significance level of 25%.

However, in the multivariable binary logistic regression model, only work experience, adverse event reports, work area/unit, and participation in patient safety programs were found to be significantly associated with patient safety culture in regional public hospitals at a significance level of 5%.

After adjusting for other factors, it was observed that compared to healthcare professionals with less than 5 years of experience, those with between 6 and 10 years of work experience had 1.81 times greater odds of having a good patient safety culture (AOR = 1.81, 95% CI = 1.13–2.88). Similarly, healthcare professionals with more than 11 years of work experience had 3.49 times greater odds of having a good patient safety culture than those with less than five years of experience (AOR = 3.49, 95% CI = 1.27–9.56).

The odds of good patient safety culture were 2.47 times greater among healthcare professionals reporting adverse events than among those who did not (AOR = 2.47, 95% CI = 1.37–4.45).

Compared with those working in medical wards, healthcare professionals working in obstetrics and pediatric wards were 53% and 79% less likely to have good patient safety (AOR = 0.47, 95% CI = 0.23–0.94) and (AOR = 0.21, 95% CI = 0.09–0.44) respectively.

Regarding participation in patient safety programs, the odds of good patient safety culture were 3.64 times greater among health care professionals who participated in such programs than among those who did not (AOR = 3.64, 95% CI = 1.91–6.92). (Table 4 shows the multivariate analysis of factors)

**Table 4:** Multivariable analysis of factors associated with patient safety culture (*N* = 461)

Variables	category	Patient safety culture		COR(95%CI)	AOR(95%CI)	*P* -value
		good	poor			
Work experience	<=5	106	160	1	1	
	6–10	94	72	2.12(1.43–3.15)	1.81(1.13–2.88)	0.013*
	>=11	22	7	4.74(1.96–11.49)	3.49(1.27–9.56)	0.015*
Adverse event report	No	164	206	1	1	
	Yes	61	30	2.55(1.58–4.14)	2.47(1.37–4.45)	0.003*
Work area/unit	Medicine	44	45	1	1	
	Surgery	27	37	1.34(0.70–2.56)	1.13(0.56–2.31)	0.734
	Obstetrics	47	22	0.46(0.24–0.88)	0.47(0.23–0.94)	0.034*
	Pediatrics	60	16	0.26(0.13–0.52)	0.21(0.09–0.44)	0.001*
	Emergency	7	35	4.88(1.97–12.17)	3.35(1.27–8.83)	0.014*
	ICU	8	23	2.81(1.14–6.95)	2.51(0.96–6.58)	0.060
	Pharmacy	18	18	0.99(0.45–2.12)	0.84(0.36–1.95)	0.685
	Laboratory	17	11	0.63(0.27–1.50)	0.55(0.21–1.42)	0.216
	Others	8	18	2.20(0.87–5.58)	1.14(0.39–3.32)	0.805
Educational status	Bachelor’s degree	114	138	1	1	
	Medical doctor	24	38	0.77(0.43–1.35)	0.72(0.37–1.39)	0.324
	master’s degree	75	50	1.82(1.18–2.81)	0.99(0.59–1.69)	0.990
	Other	12	10	1.45(0.61–3.49)	0.74(0.27–2.04)	0.564
Patient safety training	no	153	175	1	1	
	yes	72	61	1.35(0.90–2.02)	1.36(0.85–2.17)	0.206
Participate in patient safety program	no	168	219	1	1	
	yes	57	17	4.37(2.45–7.79)	3.64(1.91–6.92)	0.001*

1 = reference, others of work area = psychiatry, ICU, radiology, anesthesiology, others education status = resident, specialist, * = Significant at *P*-value ≤ 0.05, COR Crude Odd Ratio, CI Confidence Interval

## Discussion

In this study, only 48.8% of the participants had a good patient safety culture. This is lower than the 75% standard set by HSOPSC [20]. This percentage is lower than that reported in studies in Bahir Dar [21], Sarawak [22], Saudi Arabia [23], Ghana [24], and South India [25], where a prevalence of 50.8% − 74.66% is reported. On the other hand, this finding is greater than that reported in studies in the Jimma zone, Addis Ababa, Bale zone, Gondar, and Amhara region hospitals where a prevalence of 44%− 46.7% was reported [15–17, 26, 27]. This difference might arise from healthcare settings that have limited resources and may face challenges in providing optimal care and implementing safety protocols. This can result in shortcuts, increased workload, and potential safety lapses. Other possible reasons might be differences in the organizational structure, leadership, and management structure of hospitals.

Among the twelve dimensions of patient safety culture, teamwork within the unit (77.7%) was the highest contributing dimension for overall good patient safety culture. This finding is comparable to those of studies conducted in Dessie, Dilla, and the Bale Zone [16, 18, 28]. On the other hand, the frequency of event reporting (44.7%) was the least contributing dimension [18].

According to the multivariable regression, work experience, adverse event reports, work area/unit, and participation in patient safety programs were found to be significantly associated with the patient safety culture of regional public hospitals.

Accordingly, after adjusting for other covariates, the odds of good patient safety culture were greater among those who had between 6 and 10 years of experience than among those who had less than 5 years of work experience. This finding could be attributed to patient safety being increasingly recognized as a result of healthcare systems evolution and learning from past experiences. Over time, healthcare facilities have adopted various measures and programs to enhance patient safety, such as implementing standard protocols, strengthening communication and collaboration among healthcare practitioners, and cultivating a culture of accountability and transparency. Greater emphasis has been placed on patient safety and a more comprehensive culture is being developed, as rekindled through these efforts. A good patient safety culture is boosted by the increased commitment of healthcare providers and organizations to improving patient security. This leads to a positive outcome. This study is also similar to the study conducted in Iran [29].

This study revealed that there were greater odds of having a good patient safety culture among healthcare professionals who reported adverse events. This could be because when adverse events are reported, healthcare associations can identify and analyze implicit pitfalls and areas for improvement in patient care. By encouraging and promoting a culture of reporting, healthcare providers can learn from these events and make necessary changes to help prevent similar incidents in the future. This visionary approach to relating to and addressing patient safety effects fosters a culture of constant learning and improvement, ultimately benefiting patient safety. This is consistent with previous studies conducted in Amhara [26].

The findings of this study indicated that participating in patient safety programs is significantly associated with patient safety culture. This could be because these programs provide education and training on best practices and evidence-based guidelines for patient safety. By equipping healthcare providers with the knowledge and skills to identify and mitigate risks, they are better prepared to deliver safe and high-quality care. This study is also similar to a study conducted in Bahir Dar [21].

In this study, healthcare professionals who were working in pediatrics and obstetrics wards/units had lower odds of having a good patient safety culture than did those working in medical wards. The possible reason is the complexity and high-risk nature of the patient population in these specialties. Pediatrics and obstetrics involve caring for vulnerable populations, such as infants, children, and pregnant women, where even small errors can have significant consequences. This heightened level of risk can create additional stress and pressure on healthcare providers, potentially impacting their ability to prioritize patient safety. This is supported by a study conducted in Dessie [18].

Healthcare professionals working in emergency wards were more likely to have a good patient safety culture than those working in medical wards. This is consistent with the findings of previous studies conducted in Gondar [27]. This might be because patients in emergency wards have shorter stays, and they have critical cases that require close follow-up.

### Limitation of the study

- The data were collected through a self-administered questionnaire, rather than through real observation in practice.
- There is a possibility of social-desirability bias and there may be the possibility of overreporting.
- The cross-sectional study design does not establish a cause and effect relationship between the identified factors and patient safety culture.
- The results may not be generalizable to private facilities.

Furthermore, our study did not include verification of findings through administrative/management attitudes toward patient safety, which could have improved the strength of the conclusions from this study.

## Conclusion

The levels of good patient safety culture in regional public hospitals are below the standards (HSOPSC). All dimensions of patient safety culture are below the standards except for teamwork within the unit. Work experience, adverse event reporting, work area/unit, and participation in patient safety programs were found to be significantly associated with patient safety culture in regional public hospitals.

### Electronic supplementary material

Below is the link to the electronic supplementary material.

Supplementary Material 1

## Data Availability

The datasets used and analysed during the current study are available from the corresponding author on reasonable request.

## Abbreviations

AOR: Adjusted Odds Ratio
CI: Confidence Interval
COR: Crude Odds Ratio
ECMO: Extracorporeal Membrane Oxygenation
HSOPSC: Hospital Survey on Patient Safety Culture
IQR: Inter quartile range
NQSS: Ethiopian National Healthcare Quality and Safety Strategy
SPSS: Statistical Package for the Social Sciences
VIF: Variance Inflation Factors
WHO: World Health Organization
