# Supplementary material for: Patient safety culture and associated factors of regional public hospitals in Addis Ababa
Source: BMC Health Serv Res. 2024 Jul 12;24:811. doi: 10.1186/s12913-024-11262-y (PMC11245765; doi:10.1186/s12913-024-11262-y)
Supplement: Supplementary file 1 — Supplementary Material 1 [file 12913_2024_11262_MOESM1_ESM.docx]

**Hospital Survey on Patient Safety**

**Instructions**

**This survey asks for your opinions about patient safety issues, medical error, and event reporting in your hospital and will take about 10 to 15 minutes to complete.**

**If you do not wish to answer a question, or if a question does not apply to you, you may leave your answer blank.**

• *An* ***“event”*** *is defined as any type of error, mistake, incident, accident, or deviation, regardless of whether or not it results in patient harm.*

• ***“Patient safety”*** *is defined as the avoidance and prevention of patient injuries or adverse events resulting from the processes of health care delivery.*

## Annex-I: Questionary in English version

**Part I: Background information of the participants**

| S.N | Questions | Response |
| --- | --- | --- |
| 101 | How old are you? | years |
| 102 | Sex | a. Male b. Female |
| 103 | Marital status | a. single b. married  c. divorced d. widowed |
| 104 | Educational status | a. diploma b. bachelor degree  c. medical doctors d. master’s degree  e. resident physician f. specialist  g. other ………. |
| 105 | Where do you work? | a. Zewditu hospital  b. Rasdesta hospital |
| 106 | Do you have patient safety training? | a. no  b. yes |
| 107 | Do you participate in patient safety program? | a. no  b. yes |
| 108 | What is your primary work unit/ department or clinical area of the hospitals where you spent most of the work time or provides most of the clinical service? | a. Many different work area/No specific  unit  b. Medicine/non-surgical  c. Surgery  d. Obstetrics  e. Pediatrics  f. Emergency/OPD  g. Psychiatry/mental health  h. Rehabilitation  i. Pharmacy  j. Laboratory  k. Radiology  l. Anesthesiology  m. Other |
| 109 | What is your staff position in this hospital? Select ONE answer that best describes your staff position. | 1. Nurse  2. clinical nurse  3. Physical, Occupational, or Speech Therapist  4. medical doctor  5. Resident  6. pharmacist  7. dietician  8. health officer  9. Radiology Technician  10. Anesthetist  11. Lab technician  12. Administration/Management  13. Midwife  14. others  12. Others |
| 110 | How long have you worked in your current specialty or profession? | years |
| 111 | How long have you worked in this hospital? | years |
| 112 | How long have you worked in your current hospital work area/unit? | years |
| 113 | Typically, how many hours per week do you work in this hospital? | hours |
| 114 | In your staff position, do you typically have direct interaction or contact with patients? | a. YES, I typically have direct  Interaction or contact with patients.  b. NO, I typically do NOT have direct interaction or contact with patients |

**Part II: Patient Safety culture dimensions**

| **SECTION A: Your Work Area/Unit**  Please indicate your agreement or disagreement with the following statements about your work area/unit.  ***1- Strongly Disagree, 2- Disagree, 3- Neutral, 4- Agree, 5-Strongly Agree*** | | | | | | |
| --- | --- | --- | --- | --- | --- | --- |
| **I. Teamwork within units/departments** | | | | | | |
| 201 | People support one another in this unit | 1 | 2 | 3 | 4 | 5 |
| 202 | When a lot of work needs to be done quickly, we work together  as a team to get the work done. | 1 | 2 | 3 | 4 | 5 |
| 203 | In this unit, people treat each other with respect. | 1 | 2 | 3 | 4 | 5 |
| 204 | When one area in this unit gets really busy, others help out. | 1 | 2 | 3 | 4 | 5 |
| **II. Staffing** | | | | | | |
| 205 | We have enough staff to handle the workload. | 1 | 2 | 3 | 4 | 5 |
| 206 | Staff in this unit work longer hours than is best for patient care.  (negatively worded) | 1 | 2 | 3 | 4 | 5 |
| 207 | We use more agency/temporary staff than is best for patient care.  (negatively worded) | 1 | 2 | 3 | 4 | 5 |
| 208 | We work in "crisis mode" trying to do too much, too quickly.  (negatively worded) | 1 | 2 | 3 | 4 | 5 |
| **III. Organizational Learning—Continuous Improvement** | | | | | | |

| 209 | We are actively doing things to improve patient safety. | 1 | 2 | 3 | 4 | 5 |
| --- | --- | --- | --- | --- | --- | --- |
| 210 | Mistakes have led to positive changes here. | 1 | 2 | 3 | 4 | 5 |
| 211 | After we make changes to improve patient safety, we evaluate  their effectiveness. | 1 | 2 | 3 | 4 | 5 |
| **IV. Non punitive Response to Errors** | | | | | | |
| 212 | Staff feels like their mistakes are held against them. (negatively  worded) | 1 | 2 | 3 | 4 | 5 |
| 213 | When an event is reported, it feels like the person is being written  up, not the problem. (negatively worded) | 1 | 2 | 3 | 4 | 5 |
| 214 | Staff worry that mistakes they make are kept in their personnel  file. (negatively worded) | 1 | 2 | 3 | 4 | 5 |
| **V. Overall Perceptions of Patient Safety** | | | | | | |
| 215 | Patient safety is never sacrificed to get more work done. | 1 | 2 | 3 | 4 | 5 |
| 216 | Our procedures and systems are good at preventing errors from  happening. | 1 | 2 | 3 | 4 | 5 |
| 217 | It is just by chance that more serious mistakes don't happen  around here. (negatively worded) | 1 | 2 | 3 | 4 | 5 |
| 218 | We have patient safety problems in this unit. (negatively worded | 1 | 2 | 3 | 4 | 5 |
| **SECTION B: Your Supervisor/Manager**  Please indicate your agreement or disagreement with the following statements about your immediate supervisor/manager or person to whom you directly report  ***1- Strongly disagree, 2- Disagree, 3- Neutral, 4- Agree, 5- Strongly Agree*** | | | | | | |
| VI. **Supervisor/Manager Expectations & Actions Promoting Patient Safety** | | | | | | |
| 301 | My supervisor/manager says a good word when he/she sees a job  done according to established patient safety procedures. | 1 | 2 | 3 | 4 | 5 |
| 302 | My supervisor/manager seriously considers staff suggestions for  improving patient safety. | 1 | 2 | 3 | 4 | 5 |
| 303 | Whenever pressure builds up, my supervisor/manager wants us  to work faster, even if it means taking shortcuts. (negatively worded) | 1 | 2 | 3 | 4 | 5 |
| 304 | My supervisor/manager overlooks patient safety problems that  happen over and over. (negatively worded) | 1 | 2 | 3 | 4 | 5 |

| **SECTION C: Communications**  How often do the following things happen in your work area/unit?  ***1- Never, 2- Rarely, 3-Sometimes, 4- Most of the time, 5- Always*** | | | | | | |
| --- | --- | --- | --- | --- | --- | --- |
| **VII. Communication Openness** | | | | | | |
| 401 | Staff will freely speak up if they see something that may  negatively affect patient care. | 1 | 2 | 3 | 4 | 5 |
| 402 | Staff feel free to question the decisions or actions of those with  more authority. | 1 | 2 | 3 | 4 | 5 |
| 403 | Staff are afraid to ask questions when something does not seem  Right. (negatively worded) | 1 | 2 | 3 | 4 | 5 |
| **III. Feedback & Communication About Error** | | | | | | |
| 404 | We are given feedback about changes put into place based on  event reports. | 1 | 2 | 3 | 4 | 5 |
| 405 | We are informed about errors that happen in this unit. | 1 | 2 | 3 | 4 | 5 |
| 406 | In this unit, we discuss ways to prevent errors from happening  again. | 1 | 2 | 3 | 4 | 5 |
| **SECTION D: Frequency of Events Reported**  In your hospital work area/unit, when mistakes happen, how often are they reported?  ***1- Never, 2- Rarely, 3-Sometimes, 4- Most of the time, 5- Always*** | | | | | | |
| **IX. Frequency of Events Reported** | | | | | | |
| 501 | When a mistake is made, but is caught and corrected before  affecting the patient, how often is this reported? | 1 | 2 | 3 | 4 | 5 |
| 502 | When a mistake is made, but has no potential to harm the patient,  how often is this reported? | 1 | 2 | 3 | 4 | 5 |
| 503 | When a mistake is made that could harm the patient, but does  not, how often is this reported? | 1 | 2 | 3 | 4 | 5 |
| **SECTION E: Your Hospital**  Please indicate your agreement or disagreement with the following statements about your hospital  ***1- Strongly Disagree, 2- Disagree, 3- Neutral, 4-Agree, 5-Strongly Agree*** | | | | | | |
| **X. Management Support for Patient Safety** | | | | | | |

| 601 | Hospital management provides a work climate that promotes  patient safety. | | 1 | 2 | 3 | 4 | 5 |
| --- | --- | --- | --- | --- | --- | --- | --- |
| 602 | The actions of hospital management show that patient safety is a  top priority. | | 1 | 2 | 3 | 4 | 5 |
| 603 | Hospital management seems interested in patient safety only  after an adverse event happens. (negatively worded) | | 1 | 2 | 3 | 4 | 5 |
| **XI. Teamwork Across Units** | | | | | | | |
| 604 | There is good cooperation among hospital units that need to work  together. | | 1 | 2 | 3 | 4 | 5 |
| 605 | Hospital units work well together to provide the best care for  patient | | 1 | 2 | 3 | 4 | 5 |
| 606 | Hospital units do not coordinate well with each other. (negatively | | 1 | 2 | 3 | 4 | 5 |
| 607 | It is often unpleasant to work with staff from other hospital units.  (negatively worded) | | 1 | 2 | 3 | 4 | 5 |
| **XII. Handoffs & Transitions** | | | | | | | |
| 608 | Things "fall between the cracks" when transferring patients from  one unit to another. (negatively worded) | | 1 | 2 | 3 | 4 | 5 |
| 609 | Important patient care information is often lost during shift  changes. (negatively worded) | | 1 | 2 | 3 | 4 | 5 |
| 610 | Problems often occur in the exchange of information across  hospital units. (negatively worded) | | 1 | 2 | 3 | 4 | 5 |
| 611 | Shift changes are problematic for patients in this hospital.  (negatively worded) | | 1 | 2 | 3 | 4 | 5 |
| **SECTION F: Patient Safety Grade**  For item, please circle the single most appropriate latter  ***A- Excellent, B- Very Good, C- Acceptable, D- Poor, E-Failing*** | | | | | | | |
| 701 | Please give your work area/unit in this hospital an overall grade  on patient safety. | | A | B | C | D | E |
| **SECTION G: Number of Events Reported** | | | | | | | |
| 801 | In the past 12 months, did have you filled out and submitted event reports? | a. yes  b. no | | | | | |

## Annex-II: Questionnaire in Amharic Version

**1.** የመልስ ሰጪው አጠቃላይ መረጃ

| ኮድ | ጥያቄዎች | መልስ | |
| --- | --- | --- | --- |
| 101 | ዕድሜዎት ስንት ነው? | ዓመት | |
| 102 | ፆታዎ/ት ምንድንነወ? | ሀ. ወንድ  ለ. ሴት | |
| 103 | የጋብቻ ሁኔታ | ሀ. ያላገባ ለ. ያገባ  ሐ. የተፋታ መ. በሞት ያጣ | |
| 104 | የትምህርት ደረጃ | ሀ. ዲፕሎማ ለ. ባችለር ድግሪ  ሐ. ሜዲካል ዶክተር መ. የሁለተኛ ድግሪ  ሰ. ሪዝደት ሀኪም ረ. ስፔሻሊስት  ሠ. ሌላ ካለ . | |
| 105 | የስራ ቦታ - | ሀ. ዘውዲቱ ሆስፒታል  ለ. ራስ ደስታ ሆስፒታል | |
| 106 | ስለ ታካሚ ደህንነት ስልጠና ወስደህ/ሽ/ ታዉቃለህ/ሽ/? | ሀ. ወስጄ አላቅም  ለ. አዎ ወስጄ አቃለሁ | |
| 107 | በታካሚ ደህንነት ላይ ሰርተህ ታቃለህ/ሽ/? | ሀ. ሰርቸ አላቅም  ለ. ሰርቸ አቃለሁ | |
| 108 | በሆስፕታሉ ዉስጥ በየትኛዉ ክፍል ነዉ የምሰሩት፤ አገልግሎት  የምሰጡት? | 1. በተለያዩ ቦታዎች፤ ቋም ቦታ የለኝም  2. ሜድካል ዋርድ  3. ሰርጂካል ዋርድ  4. ኦብስታትርክስ ዋርድ  5. ፐዲያትሪክስ ዋርድ  6. ድንገተኛ ክፍል  7. ሳይካትር ዋርድ  8. ማገገምያ ክፍል  9. ፋርማሲ ክፍል  10. ላብራቶሪ ክፍል  11. ራድዮሎጂ ክፍል  12. አንስተዥያ ክፍል  13. ለላ ካለ | |
| 109 | በዚህ ሆስፒታል የስራ ሙያዎ ምንድንነው? | 1. ነርስ  2. ክልኒካል ነርስ  3. የአካላዊ ፣ የሙያዊ ወይም የንግግር ቴራፒስት  4. ሐኪም  5. ሪዝደት ሀኪም  6. ፋርማሲስት  7. የስነ ምግብ ባለሙያ  8. ጤና መኮንን  9. ራድዮሎጂ ባለሙያ  10. አንስተዥያ ባለሙያ  11. ላብራቶሪ ባለሙያ  12. የአስተዳዳር ክፍል  13. ምድዋይፈሪ  14. ሌላ (ይገለጽ) | |
| 110 | በሙያዎ ምን ያክል ጊዜ አገልግለዋል? | ዓመት | |
| 111 | ምን ያክል ጊዜ በዚህ ሆስፒታል አገልግለዋል? | ዓመት | |
| 112 | | አሁን ባሉበት የስራ ክፍል/ኬዝቲም ለምን ያህል ጊዜ አገልግለዋል? | ዓመታት |
| 113 | | በሆስፒታለ በ1 ሳምንት ውስጥ ስንት ሰዓታት ያገለግላሉ? | ሰዓታት |
| 114 | | በምሰሩበት ክፍል ዉስጥ ከበሽተኛ ጋር ቀጥተኛ የሆነ ግንኙነት  ወይም ንክክ አልዎት? | ሀ. አዎ አለኝ  ለ. አይ የለኝም |

ክፍል**-2** ተሳታፍዉ በሆስፕታሉ ዉስጥ ስላለዉ የታካምዎች ደህንነት ባህል ያለዉ አመለካከት

| ሀ. የምሰሩበትን ክፍል በተመለከተ  እባክዎትን ከታች ባለት እርስዎ ስለምሰሩበት ሆስፕታል በተገለፁ ሀሳቦች መስማማት/አለመስማማትዎን ይጠቁሙ ለእያንዳንዱ ጥያቄ የሚስማማዎትን ያክብቡ  1- በጣም አልስማማም ***2-*** አልስማማም ***3-*** ገለልተኛ ***4-*** እስማማለሁ ***5-*** በጣም እስማማለሁ | | | | | | |
| --- | --- | --- | --- | --- | --- | --- |
| **I. Teamwork within units/departments (**በስራ ክፍላችን ተባብረን የመስራት ሁኔታ) | | | | | | |
| 201 | በስራ ክፍላችን እርስበርስ እንደጋገፋለን | 1 | 2 | 3 | 4 | 5 |
| 202 | የስራ ጫና ሲኖር በጋራ ተባብረን እንሰራለን | 1 | 2 | 3 | 4 | 5 |
| 203 | በስራ ክፍላችን ተከባብረን እንሰራለን | 1 | 2 | 3 | 4 | 5 |
| 204 | በተቋማችን በሌላ ስራ ክፍል ክፍተት ሲኖር እንተጋገዛለን | 1 | 2 | 3 | 4 | 5 |
| **II. Staffing (**የሰው ሃይልን በተመለከተ**)** | | | | | | |
| 205 | የስራ ጫናውን ለመቋቋም የሚያስችል በቂ የሰው ሃይል አለን | 1 | 2 | 3 | 4 | 5 |
| 206 | በዚህ ክፍል ውስጥ ያሉ ሰራተኞች ለታካሚው ከሚያስፈልገው ሰዓት በላይ ይሰራሉ*** | 1 | 2 | 3 | 4 | 5 |
| 207 | ጊዜያዊ ሰራተኞች መጠቀም ለታካሚዎች የተሸለ ደህንነት ጥሩ ነው*** | 1 | 2 | 3 | 4 | 5 |
| 208 | ብዙ ስራ በፍጥነት ለመስራት ጫና ውስጥ እንገባለን*** | 1 | 2 | 3 | 4 | 5 |
| **III. Organizational Learning—Continuous Improvement (**ተቋማዊ ለውጥን በተመለከተ**)** | | | | | | |
| 209 | የታካሚዎችን ደህንነት ለማሻሸል በንቃት እየሰራን ነው | 1 | 2 | 3 | 4 | 5 |
| 210 | ግድፈቶች/ስህተቶች ለአወንታዊ ለውጦች ያመሩናል | 1 | 2 | 3 | 4 | 5 |
| 211 | የታካሚዎችን ደህንነት ለማሻሸል ለውጥ ካደረግንም በኋላ ውጤታማነቱንም  እንገመግማለን | 1 | 2 | 3 | 4 | 5 |
| **IV. Non punitive Response to Errors (**ቅጣት የሌለበት ግብረ መልስ ስለመሰጠቱ**)** | | | | | | |
| 212 | ሰራተኞቹ ስህተቶቻቸው የሚያስወቅሳቸው መስሎ ይሰማቸዋል*** | 1 | 2 | 3 | 4 | 5 |
| 213 | አንድ ድርጊት ሲፈጸም ለችግሩ መፍትሔ ከመስጣት ይልቅ የችግሩ ፈጣሪ ግለሰብ  ተወቃሽ መስሎ ይሰማናል*** | 1 | 2 | 3 | 4 | 5 |
| 214 | ሰራተኞቻችን ስህተቶቻቸው በማህደራቸው የሚቀመጥ ይመስላቸዋል*** | 1 | 2 | 3 | 4 | 5 |
| **V. Overall Perceptions of Patient Safety (**አጠቃላ የታካሚወች ደህንነት በተመለከተ**)** | | | | | | |
| 215 | የታካሚዎች ደህንነት ብዙ ስራ ለመሥራት ሲባል አደጋ ላይ አይወድቅም | 1 | 2 | 3 | 4 | 5 |

| 216 | የስራ ሂደታችንና ደንባችን ችግሮች እንዳይፈጠሩ ለማድረግ ጥሩ ናቸው | 1 | 2 | 3 | 4 | 5 |
| --- | --- | --- | --- | --- | --- | --- |
| 217 | እዚህ የጤና ችግር ያልተከሰተው የአጋጣሚ ጉዳይ ሆኖ ነው*** | 1 | 2 | 3 | 4 | 5 |
| 218 | በዚህ ክፍል ውስጥ የታካሚ ደህንነት ችግሮች አሉብን*** | 1 | 2 | 3 | 4 | 5 |
| ለ**.** የቅርብ አለቃዎን በተመለከተ  እባክዎትን ከታች ባለት የቅርብ አለቃዎን በተመለከተ በተገለፁ ሀሳቦች መስማማት/አለመስማማትዎን ይጠቁሙ  ***1.*** በጣም አልስማማም ***2-*** አልስማማም ***3-*** ገለልተኛ ***4-*** እስማማለሁ ***5-*** በጣም እስማማለሁ | | | | | | |
| **VI. Supervisor/Manager Expectations & Actions Promoting Patient Safety (**አለቃዎን  በተመለከተ) | | | | | | |
| 301 | አለቃዬ የታካሚን ደህንነት በጠበቀ መልኩ ስራ ሲተገበር ደስ ይለዋል | 1 | 2 | 3 | 4 | 5 |
| 302 | አለቃዬ ከሰራተኞቹ የሚመጡትን የታካሚዎችን ደህንነት ለማስጠበቅ የሚረዱ  ሀሳቦችን ይቀበላል | 1 | 2 | 3 | 4 | 5 |
| 303 | አለቃዬ የስራ ጫና በሚኖርበት ጊዜ አቋራጭ መንገዶችን በመጠቀምም ቢሆን ስራው ቶሎ እንዲሰራ ይፈልጋል*** | 1 | 2 | 3 | 4 | 5 |
| 304 | አለቃዬ በታካሚዎች ደህንነት ላይ የሚፈጠሩ ችግሮችን ችላ ይላል*** | 1 | 2 | 3 | 4 | 5 |
| ሐ. ዉይይትን በተመለከተ  ከዚህ ቀጥሎ የሚቀርቡ ጥያቄዎች ምን ያህል ጊዜ በሆስፕታላችሁ ይከሰታሉ? ለእያንዳንዱ ጥያቄ የሚስማማዊትን ያክብቡ  ***1.*** ምንም ***2-***  አልፎአልፎ ***3-*** ኣንዳንድ ጊዜ ***4-*** ብዙውን ጊዜ ***5-*** ሁል ጊዜ | | | | | | |
| **VII. Communication Openness (**በግልጽ ስለመነጋገር**)** | | | | | | |
| 401 | ሰራተኞቻችን የታካሚዎችን አገልግሎት የሚጎዳ ነገር ባዩ ጊዜ በነጻነት ይገልጻሉ | 1 | 2 | 3 | 4 | 5 |
| 402 | ሰራተኞቻችን በኃሊፊዎች ውሳኔ ወይም ድርጊት ያልገባቻውን በነጻነት ይጠይቃሉ | 1 | 2 | 3 | 4 | 5 |
| 403 | ሰራተኞች አንድ ነገር ትክክል መስሎ በማይታይበት ጊዜ ጥያቄዎችን ለመጠየቅ ይፈራሉ *** | 1 | 2 | 3 | 4 | 5 |
| **III. Feedback & Communication About Error (**ስህተቶች ሲከሰቱ ግብረመልስ ስለመስጠት እና  ስለመወያየት**)** | | | | | | |
| 404 | በስራችን ስለመጣው ለውጥ ግብረመልስ ይሰጠናል | 1 | 2 | 3 | 4 | 5 |
| 405 | በስራ ክፍላችን ስህተት ሲከሰት እንድናውቅ ይደረጋል | 1 | 2 | 3 | 4 | 5 |
| 406 | በስራ ክፍላችን ስህተት ዳግም እንዳይፈጠሩ መከላከያ መንገዶችን እንወያየለን | 1 | 2 | 3 | 4 | 5 |
| መ. የችግሮች ሪፖርት ብዛት  ከዚህ ቀጥሎ የሚቀርቡ ጥያቄዎች ምን ያህል ጊዜ በሆስፕታላችሁ ይከሰታሉ?  1. ምንም ***2-***  አልፎአልፎ ***3-*** ኣንዳንድ ጊዜ ***4-*** ብዙውን ጊዜ ***5-*** ሁል ጊዜ | | | | | | |
| **IX. Frequency of Events Reported** | | | | | | |
| 501 | የተፈጠረው ስህተት በህመምተኛው ላይ ጉዳት ከማድረሱ በፊት ቢታወቅ እና  እርማት ቢደረግ ምን ያህል ሪፖርት ይደረጋሉ? | 1 | 2 | 3 | 4 | 5 |

| 502 | ሰህተት ቢፈጠር እና ህመምተኞችን የማይጎዳ ቢሆን እንኳ ምን ያህል ሪፖርት  ይደረጋሉ? | | 1 | 2 | 3 | 4 | 5 |
| --- | --- | --- | --- | --- | --- | --- | --- |
| 503 | ህመምተኞችን የሚጎዳ ስህተት ቢፈጠር ጉዳት ባያደርስ እንኳን ምን ያህል ሪፖርት  ይደረጋሉ? | | 1 | 2 | 3 | 4 | 5 |
| ሠ. ስለ ሆስፒታልዎ  እባክዎትን ከታች ባለት እርስዎ ስለምሰሩበት ሆስፕታል በተገለፁ ሀሳቦች መስማማት/አለመስማማትዎን ይጠቁሙ ለእያንዳንዱ ጥያቄ የሚስማማዎትን ያክብቡ  ***1.*** በጣም አልስማማም ***2-*** አልስማማም ***3-*** ገለልተኛ ***4-*** እስማማለሁ ***5-*** በጣም እስማማለሁ | | | | | | | |
| **X. Management Support for Patient Safety (**የሆስፒታሉ አመራር ለታካሚዎች ደህንነት የሚያደርገው  ድጋፍ**)** | | | | | | | |
| 601 | የሆስፒታሉ አስተዳደር የታካሚችን ደህንነት የሚያበረታታ ምቹ የስራ ሁኔታ  ያመቻቸል | | 1 | 2 | 3 | 4 | 5 |
| 602 | የአስተዳደሩ ድርጊቶች ለታካሚዎች ደህንነት ቅድሚያ መሰጠቱን ያሳያል | | 1 | 2 | 3 | 4 | 5 |
| 603 | አስተዳደሩ ስለታካሚዎች ደህንነት የሚያነሳው ችግሮች ከተከሰቱ በኋላ ነው*** | | 1 | 2 | 3 | 4 | 5 |
| **XI. Teamwork Across Units (**የሆስፒታሉ ሰራተኞች ከሌላ የስራ ክፍል ጋር አብረው **)** | | | | | | | |
| 604 | በሆስፒታሉ የስራ ክፍሎች ጥሩ የሆነ ተባብሮ የመስራት ሁኔታአለ | | 1 | 2 | 3 | 4 | 5 |
| 605 | ለታካሚዎች የተሸለ የህክምና አገልግሎት ለመስጣት ኬዝቲሞች በጋራ ይሰራሉ | | 1 | 2 | 3 | 4 | 5 |
| 606 | የሆስፒታሉ የስራ ክፍልች በቅንጅት አይሰሩም*** | | 1 | 2 | 3 | 4 | 5 |
| 607 | ከሌላ የስራ ክፍሌ/ኬዝቲም/ ሰራተኞች ጋር መስራት አይመችም*** | | 1 | 2 | 3 | 4 | 5 |
| **XII. Handoffs & Transitions (**ስለታካሚወች ዝውውር እና የሰራተኞች ቅያሪን በተመለከተ **)** | | | | | | | |
| 608 | ህመምተኞች ከአንድ የስራ ክፍል ወደሌላ ክፍል ሲዘዋወሩ ክፍተት አለ*** | | 1 | 2 | 3 | 4 | 5 |
| 609 | አስፈሊጊ የታካሚዎች መረጃ በፈረቃ ልውውጥ ጊዜ ይጠፋል*** | | 1 | 2 | 3 | 4 | 5 |
| 610 | በመረጃ ልውውጥ ጊዜ በአብዘኛው ችግር ይከሰታል*** | | 1 | 2 | 3 | 4 | 5 |
| 611 | በሆስፒታላችን የፈረቃ ልውውጥ ለታካሚዎቻችን አስቸጋሪ ነው*** | | 1 | 2 | 3 | 4 | 5 |
| ረ. **Patient Safety Grade (**የታካምዎችን ደህንነትን በተመለከተ)  ለጥያቄዉ፤ እባክዎ የምስማሙበትን መልስ የያዘዉን ፍደል ያክብቡ  ሀ***-*** እጅግ በጣም ጥሩ ለ***-*** በጣም ጥሩ ሐ***-*** ጥሩ መ***-*** ዝቅተኛ ሠ***-***በጣም ዝቅተኛ | | | | | | | |
| 701 | የምሰሩበት ሆስፕታል አጠቃላይ የታካምዎች ደህንነት አጠባበቅ ምን ይመስላል? | | ሀ | ለ | ሐ | መ | ሠ |
| ሰ. **Events Reported (**ሪፖርት የተደረጉ ክስተቶች ብዛት) | | | | | | | |
| 801 | ባለፉት 12 ወራት ክስተቶችን ሪፖርት አድርገዋል | ሀ. አዎ  ለ. አላደረግሁም | | | | | |
